# Quantitative Assessment of the Impact of Geometric Distortions and Their Correction on fMRI Data Analyses

**DOI:** 10.3389/fnins.2021.642808

**Published:** 2021-03-09

**Authors:** Rodolfo Abreu, João Valente Duarte

**Affiliations:** ^1^Coimbra Institute for Biomedical Imaging and Translational Research (CIBIT), Institute for Nuclear Sciences Applied to Health (ICNAS), University of Coimbra, Coimbra, Portugal; ^2^Faculty of Medicine, University of Coimbra, Coimbra, Portugal

**Keywords:** fMRI, susceptibility artifact, geometric distortions and correction, B_0_ field mapping, neuroimaging

## Abstract

Functional magnetic resonance imaging (fMRI) data is typically collected with gradient-echo echo-planar imaging (GE-EPI) sequences, which are particularly prone to the susceptibility artifact as a result of B_0_ field inhomogeneity. The component derived from in-plane spin dephasing induces pixel intensity variations and, more critically, geometric distortions. Despite the physical mechanisms underlying the susceptibility artifact being well established, a systematic investigation on the impact of the associated geometric distortions, and the direct comparison of different approaches to tackle them, on fMRI data analyses is missing. Here, we compared two different distortion correction approaches, by acquiring additional: (1) EPI data with reversed phase encoding direction (TOPUP), and (2) standard (and undistorted) GE data at two different echo times (GRE). We first characterized the geometric distortions and the correction approaches based on the estimated ΔB_0_ field offset and voxel shift maps, and then conducted three types of analyses on the distorted and corrected fMRI data: (1) registration into structural data, (2) identification of resting-state networks (RSNs), and (3) mapping of task-related brain regions of interest. GRE estimated the largest voxel shifts and more positively impacted the quality of the analyses, in terms of the (significantly lower) cost function of the registration, the (higher) spatial overlap between the RSNs and appropriate templates, and the (significantly higher) sensitivity of the task-related mapping based on the *Z*-score values of the associated activation maps, although also evident when considering TOPUP. fMRI data should thus be corrected for geometric distortions, with the choice of the approach having a modest, albeit positive, impact on the fMRI analyses.

## Introduction

The quality of magnetic resonance imaging (MRI) data depends on numerous factors, one of the most critical being the homogeneity of the static magnetic field B_0_ (Jezzard, 2012). B_0_ field inhomogeneity will induce the so-called susceptibility artifact, derived from in-plane spin dephasing (inducing pixel intensity variations and geometric distortions) and through-plane spin dephasing (inducing pixel intensity variations as well, and ultimately signal loss) (Ojemann et al., 1997). The latter is typically compensated by z-shimming the MR scanner (Zhao et al., 2005; Du et al., 2007). Regarding the effects of in-plane spin dephasing, geometric distortions are far more concerning, as they are marked by apparent shifts from one local position in the image, and possibly the stretching or compression over to a larger, or into a smaller area, respectively. Single-shot gradient-echo echo-planar imaging (GE-EPI) sequences are the most prone to geometric distortions, mainly due to the long time interval between the acquisition of adjacent *k*-space points in the phase-encoding direction, which permits a significant local phase accumulation relative to that produced by the phase-encoding gradients (Jezzard, 2012). Given their ability to acquire whole-brain volumes in a few seconds (or even faster), functional magnetic resonance imaging (fMRI) data has been collected using GE-EPI sequences for decades, and thus geometric distortions are inevitably present.

Several approaches have been proposed to reduce the time between phase-encoding steps, the most widely used being parallel imaging (PI) (Deshmane et al., 2012). PI techniques reduce the number of *k*-space lines in the phase-encoding direction that need to be acquired by the chosen acceleration factor; this minimizes the artifact, but at the cost of a loss in signal-to-noise ratio, and potential reconstruction instabilities due to the sparser sampling of *k*-space. In the case of unsuccessful distortion prevention, an imperfect registration of functional images into structural images is commonly observed, the latter typically unaffected by the field inhomogeneity. This is particularly relevant for anatomically localizing functional activations (particularly in deep brain regions), and whenever group-level analyses are conducted which require the co-registration into a common space (Hutton et al., 2002).

A plethora of methods have been proposed for geometric distortion correction. These can be roughly divided into four main categories: (1) the acquisition of several single-shot EPI scans per slice, from which the point spread function (PSF) of each voxel is mapped, allowing the estimation of the underlying displacement field (Robson et al., 1997; Zeng and Constable, 2002; Zaitsev et al., 2004); (2) the non-linear registration of distorted functional images into undistorted structural images, constraining however the non-linear transformations to the phase-encoding direction (Bhushan et al., 2015); (3) the acquisition of additional standard (i.e., with a single phase-encoding step per excitation), undistorted GE images at two different echo-times (TE), and from the phase difference between the two images, estimate the B_0_ field offset (Jezzard and Balaban, 1995; Matakos et al., 2014); and (4) the acquisition of additional EPI images with reversed phase-encoding direction, and thus with also reversed distortions, from which a displacement field is estimated under the assumption that the undistorted image is midway between the two distorted images (Andersson et al., 2003; Hedouin et al., 2017). Mainly motivated by their reasonable scanning times and computational efficiency, the last two categories are by far the most commonly used for geometric distortion correction (Holland et al., 2010; Jezzard, 2012; Graham et al., 2017). Importantly, only a few studies have explicitly investigated the impact of these distortions on fMRI data analyses, and correcting them through the acquisition of additional GE images at different echo-times. The first study showed that the statistical power of a group analysis of subjects performing motor and auditory tasks is improved upon distortion correction (Cusack et al., 2003). More recently, it has also been shown that with the correction for the distortions, functional connectivity of resting-state networks (RSNs) comprising distorted brain areas can be measured more robustly, and that the specificity of their fluctuations in terms of reflecting neuronal activity, rather than noise, is improved (Togo et al., 2017). This is critical when conducting studies on the static and dynamic functional connectivity within- and between-networks, on both healthy and clinical populations (Di and Biswal, 2013; Preti et al., 2017; Allen et al., 2018; Du et al., 2018); performing any subsequent analyses on RSNs devoid of the associated well-known brain regions would compromise the interpretation of the results.

Despite the physical mechanisms underlying the susceptibility artifact being well established, a systematic investigation on the impact of the associated geometric distortions, and the direct comparison of different approaches to tackle them, on fMRI data analyses is missing. For that purpose, we started by collecting (f)MRI data at 3T from 20 healthy participants performing a simple visual motion functional localizer and a visual biological motion (BM) perception task. We then applied two distortion correction approaches by estimating the displacement field with two standard and undistorted GE images acquired at different TEs (field mapping approach, GRE) and with two EPI images with reversed phase-encoding directions [anterior–posterior (AP) and posterior–anterior (PA) approach, TOPUP; Andersson et al., 2003]. In order to quantitatively assess the impact of the distortions and their correction, we first characterized the geometric distortions and the correction approaches based on the estimated B_0_ field offset and voxel shift maps (VSMs), and then conducted three types of analyses on the uncorrected and distortion-corrected fMRI data: (1) estimation of the cost function from the registration between the functional and the structural images; (2) identification and characterization of group RSNs [because RSNs have been shown to be also present in task-based studies (Di et al., 2013; Cole et al., 2016)]; and (3) mapping of the brain areas related to motion perception in general, and in particular those involved in a visual BM perception task (Chang et al., 2018).

## Materials and Methods

### Participants

Twenty healthy participants (mean age: 28 ± 6 years; 11 males) were recruited. All participants had normal or corrected-to-normal vision, and no history of neurological disorders. The study was approved by the Ethics Commission of the Faculty of Medicine of the University of Coimbra, and was conducted in accordance with the declaration of Helsinki. All subjects provided written informed consent to participate in the study.

### Experimental Protocol

The imaging session was performed at the Portuguese Brain Imaging Network (Coimbra, Portugal) and consisted of five functional runs: first, a functional localizer of the human middle temporal area (hMT+/V5, a low level visual area well-known to respond to simple global motion patterns), followed by four runs of BM perception.

The localizer run consisted of 12 blocks of 28 s; each block started by a set of dots moving towards and away from a central fixation cross at a constant speed (5 deg/s) for 18 s, followed by a 10 s pattern of stationary dots. The hMT+/V5 was then identified as the region that responded significantly higher to moving dots than to static dot fields (Huk et al., 2002). Each of the four BM perception runs consisted of 10 blocks of 38 s, with each block comprising a human point-light walker facing rightward presented in a sagittal view at 60 Hz. Each stimulation block was followed by a 22 s baseline period, during which a fixation cross was displayed. For all runs, participants were instructed to watch passively but attentively.

### MRI Data Acquisition

Imaging was performed on a 3T Siemens MAGNETOM Prisma Fit MRI scanner (Siemens, Erlangen) using a 64-channel RF receive coil. In order to minimize head motion and scanner noise related discomfort, foam cushions and earplugs were used, respectively. The functional images were acquired using a 2D simultaneous multi-slice (SMS) GE-EPI sequence (3× SMS and 2× in-plane GRAPPA acceleration), with the following parameters: TR/TE = 1000/30.2 ms, voxel size = 2.5 × 2.5 × 2.5 mm^3^, 51 axial slices (no gap and whole-brain coverage), FOV = 195 × 195 mm^2^, FA = 68°, bandwidth = 2086 Hz/pixel, echo spacing (effective) = 0.57 (0.285) ms, 78 echoes per excitation pulse and phase encoding in the AP direction. The start of each trial was synchronized with the acquisition of the functional images. For each participant, 360 volumes were acquired during the localizer run, yielding 6 min of duration; the remaining four functional runs (BM perception) consisted of 600 volumes (10 min) each.

Before each functional run, additional data was collected for geometric distortion correction. First, field map imaging was performed with a double-echo spoiled GE sequence, with the following parameters: TR = 400 ms, TE1/TE2 = 4.92/7.38 ms (difference between TEs, ΔTE, of 2.46 ms chosen for the field strength of 3T to ensure that water and lipid spins are in phase), voxel size = 2.5 × 2.5 × 2.5 mm^3^ and 51 axial slices (matching the parameters of the functional images), FOV = 192 × 192 mm^2^, FA = 60°. From this sequence, two magnitude (one at each TE) and one phase difference images were collected, from which the displacement field was calculated (GRE approach). Then, 10 volumes using the same parameters of the functional images but with reversed phase-encoding direction (PA) were collected, from which the displacement field was estimated (TOPUP approach; Andersson et al., 2003).

A T_1_-weighted, magnetization-prepared rapid acquisition gradient-echo (MPRAGE) sequence was used to collect structural data (1 mm isotropic, 192 slices, TR/TE = 2530/3.5 ms, 2× in-plane GRAPPA acceleration), allowing for the subsequent co-registration of the functional data.

### Data Pre-processing

**FIGURE 1:**
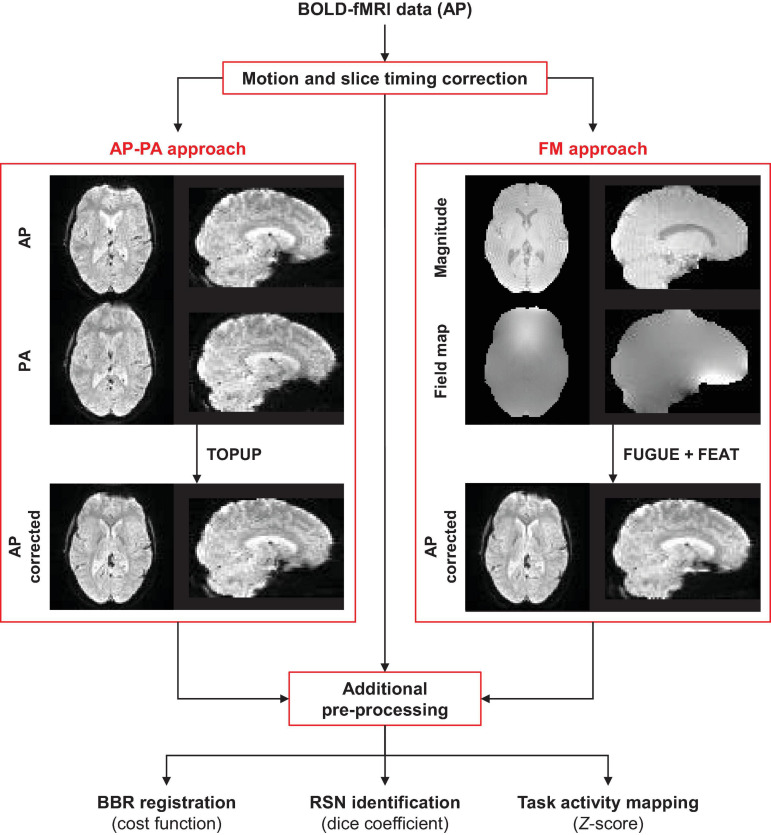
Schematic diagram of the processing pipeline. The fMRI data of each functional run (collected with the AP phase encoding direction) is first submitted to motion and slice timing correction. Then, geometric distortions are corrected using TOPUP (requires one AP and one PA functional image) or GRE (requires a magnitude and a displacement field converted to radian/s) approaches. Subsequently, additional pre-processing steps are performed on the corrected and uncorrected data, followed by the three different data analyses: (1) registration into structural data, (2) identification of resting-state networks, and (3) mapping of task-related brain regions of interest.

The main steps of the processing pipeline (described here and in the next section) proposed in this work for correcting geometric distortions and quantifying their impact on fMRI data analyses, are depicted in Figure 1.

#### fMRI Data Cleanup

The first 10 s of data were discarded to allow the fMRI signal to reach steady-state, and non-brain tissue was removed using FSL tool BET (Smith, 2002). Subsequently, slice timing and motion correction (average head motion across subjects and runs: 0.37 ± 0.44 mm) were performed using FSL tool MCFLIRT (Jenkinson et al., 2002), followed by geometric distortion correction using the TOPUP and GRE approaches (see below). Then, nuisance fluctuations were removed, a high-pass temporal filtering with a cut-off period of 100 s was applied, and spatial smoothing using a Gaussian kernel with full width at half-maximum (FWHM) of 4 mm was performed. The nuisance fluctuations (including physiological noise) were modeled by linear regression using the following regressors (Abreu et al., 2017): (1) quasi-periodic fluctuations related to cardiac and respiratory cycles were modeled by a fourth order Fourier series using RETROICOR (Glover et al., 2000); (2) aperiodic fluctuations associated with changes in the heart rate as well as in the depth and rate of respiration were modeled by convolution with the respective impulse response functions [as described in Chang et al. (2009)]; (3) the average BOLD fluctuations in white matter (WM) and cerebrospinal fluid (CSF); (4) the six motion parameters estimated by MCFLIRT; and (5) scan nulling regressors (motion scrubbing) associated with volumes acquired during periods of large head motion – motion spikes; these were determined using the FSL utility *fsl_motion_outliers*, whereby the DVARS metric proposed in Power et al. (2012) is first computed, and then thresholded at the 75th percentile plus 1.5 times the inter-quartile range. fMRI data with all these processing steps except for the geometric distortion correction was also considered for comparison purposes.

#### Structural Data Processing

For each participant, WM and CSF masks were obtained from the respective T_1_-weighted structural image by segmentation into gray matter, WM and CSF using FSL tool FAST (Zhang et al., 2001). The functional images were linearly co-registered with the respective T_1_-weighted structural images using FSL tool FLIRT, and subsequently with the Montreal Neurological Institute (MNI) (Collins et al., 1994) template, using non-linear transformations estimated with the FSL tool FNIRT (Jenkinson and Smith, 2001; Jenkinson et al., 2002). Both WM and CSF masks were transformed into the functional space through the inverse of the previously estimated linear transformation and using nearest neighbor interpolation; these were then eroded using a 3 mm spherical kernel in order to minimize partial volume effects (Jo et al., 2010). Additionally, the eroded CSF mask was intersected with a mask of the large ventricles from the MNI space, following the rationale described in Chang and Glover (2009).

#### Geometric Distortion Correction

Regarding the TOPUP approach, the FSL tool TOPUP was used (with the standard parameters provided), which requires one AP and one PA functional image (Andersson et al., 2003). The AP image was selected as the middle volume of each functional run, which is also the reference volume for motion correction; in this way, the estimated displacement field will be alignment-wise valid for all volumes. With this and the last volume of the PA acquisition, the displacement field used for B_0_-unwarping is then estimated. As for the GRE approach, the FSL utility *fsl_prepare_fieldmap* was first used to: (1) rescale the phase difference map to values between −π and +π; (2) unwrap the scaled phase difference map using the FSL tool PRELUDE; (3) divide by ΔTE to convert to units of radian/s; and (4) smooth the resulting displacement field with FSL tool FUGUE. The smoothed field and one of the magnitude images (the latter for masking the brain) were then used as input in the B_0_-unwarping step as part of the pre-processing pipeline of the FSL tool FEAT, which also performs all the co-registrations necessary of the functional and field map images into the structural image. Despite the different steps, each approach introduced a similar amount of spatial blurring to the distortion corrected data, as the same number (three) of interpolations (i.e., transformations) were performed.

In order to characterize the displacement fields estimated by the two approaches, these were first converted from radian/s to Hz by dividing them by 2π, yielding the associated ΔB_0_ maps, which reflect the voxel-wise deviations from the true static magnetic field B_0_. From these, the VSMs describing the amount (in mm) by which each voxel should be shifted in the PE direction to regain its true position was calculated according to Dymerska et al. (2018):

$$\mathrm{VSM}=s_{\text{y}}\cdot \frac{\Delta \,{\text{B}}_{0}}{B\,W_{\text{PE}}\times R}$$

where *BW*_PE_ = 1/(*echospacing*×*n*_y_) is the bandwidth in the PE direction, with *n*_*y*_ = 78, and *s*_*y*_ = 2.5 mm the number and resolution, respectively, of voxels in this direction; and *R* = 2 the in-plane acceleration factor.

The ability of TOPUP and GRE to approximate the PA and AP images after applying the respective displacement fields was assessed by comparing these images in terms of the normalized mean squared error (nMSE) as proposed in In et al. (2017), and the cross-correlation. Additionally, the corrected PA and AP images were compared between the two approaches, also in terms of the nMSE and cross-correlation, as to investigate the consistency between TOPUP and GRE.

### fMRI Data Analyses

All the following analyses were performed on pre-processed but uncorrected fMRI data, and fully pre-processed fMRI data, the latter including the correction for geometric distortions using the TOPUP and the GRE approaches.

#### Boundary-Based Registration

As recommended in FSL tool FEAT, the co-registration of functional and field map images into structural images was performed using the boundary-based registration (BBR) method, which considers WM-driven boundaries from the structural image (Greve and Fischl, 2009). These boundaries are then mapped to the functional volume using a 6 degrees-of-freedom transformation, and the difference between the intensity of pairs of voxels transversely located at 2 mm either side of points along the WM boundaries is defined as the cost function.

The BBR cost function values were then used to quantify the quality of the registration of the functional images with and without geometric distortion correction, and across correction approaches.

#### Identification of Resting-State Networks

The pre-processed fMRI data were submitted to a group-level probabilistic spatial ICA (sICA) decomposition using the FSL tool MELODIC (Beckmann and Smith, 2004), whereby the data of each run for all participants is temporally concatenated prior to the sICA step, as recommended in the MELODIC’s guide for the identification of RSNs^1^. The optimal number of independent components (ICs) was automatically estimated based on the eigenspectrum of its covariance matrix (Beckmann and Smith, 2004), with an average of approximately 40 ICs across runs.

An automatic procedure for the identification of well-known RSNs was then applied, in which the spatial maps of the ICs (thresholded at *Z* = 3.0) were compared with those of the 10 RSN templates described in Smith et al. (2009), in terms of spatial overlap computed as the Dice coefficient (Dice, 1945). For each template, the IC map yielding the highest Dice coefficient was determined as the corresponding RSN. In the cases of non-mutually exclusive assignments, the optimal assignment was determined by randomizing the order of the RSN templates (a maximum of 10000 possible combinations were considered, for computational purposes), and then sequentially, and mutually exclusively, assigning them to the IC maps based on their Dice coefficient. The assignment with the highest average Dice coefficient across all RSN templates was then deemed optimal, yielding the final set of RSNs: three visual networks (RSN 1–3), the default mode network (DMN), (RSN4), a cerebellum network (RSN5), a motor network (RSN6), an auditory network (RSN7), the salience network (RSN8), a right language network (RSN9), and a left language network (RSN10).

The maximum average Dice coefficient values were used to quantify the ability to accurately identify RSNs with and without geometric distortion correction, and across correction approaches. For the purpose of determining how affected the RSNs were by geometric distortions, the minimum and maximum voxel shifts within each RSN were extracted from the participant-averaged VSMs (after transforming them into the MNI space using nearest neighbor interpolation), for each run separately (note that RSNs were obtained at the group level, and thus are in the MNI space as well).

#### hMT+ and BM-Related Activity Mapping

For the purpose of mapping hMT+/V5 from the localizer run, and the regions involved in the BM perception task from the other four runs, a general linear model (GLM) framework was used. For both the localizer and BM runs, a GLM comprising a single regressor was built, based on unit boxcar functions with ones during the blocks of stimulation periods, and zeros during baseline periods. The regressor was convolved with a canonical, double-gamma hemodynamic response function (HRF), and then included in the GLM that was subsequently fitted to the fMRI data using FSL tool FILM (Woolrich et al., 2001); voxels exhibiting significant changes between stimulation and baseline periods were identified by cluster thresholding (voxel *Z* > 2.5, cluster *p* < 0.05). For the localizer run, group activation maps were obtained considering only mixed effects; for the BM runs, a fixed effects analysis was first performed to create subject-specific average activation maps across the four runs, followed by a mixed effects analysis generating the overall group BM activation maps across subjects and runs. These analyses were conducted using the FSL tool FLAME (Beckmann et al., 2003).

Similarly to the RSN analysis, the degree at which the group activation maps were affected by geometric distortions was determined by extracting the minimum and maximum voxel shifts within each map from the participant-averaged VSMs in the MNI space.

#### Statistical Analysis

The main effect of performing geometric distortion correction, and the approach used for that purpose, on the difference between PA and AP images was first evaluated by means of a 1-way analysis of variance (ANOVA), considering the nMSE and cross-correlation measures separately. Then, similar 1-way ANOVA tests were also performed on each of the fMRI quality measures (the BBR cost function, the Dice coefficient of the RSNs, and the average and maximum *Z*-score of the hMT+ and BM group activation maps). For all ANOVAs, multiple comparisons using 1-way ANOVA between the correction approaches (and no correction) were performed with a *post hoc* statistical test using the Tukey-Kramer correction. A level of significance of *p* < 0.05 was considered.

## Results

### Characterization of Geometric Distortions and Correction Approaches

**FIGURE 2:**
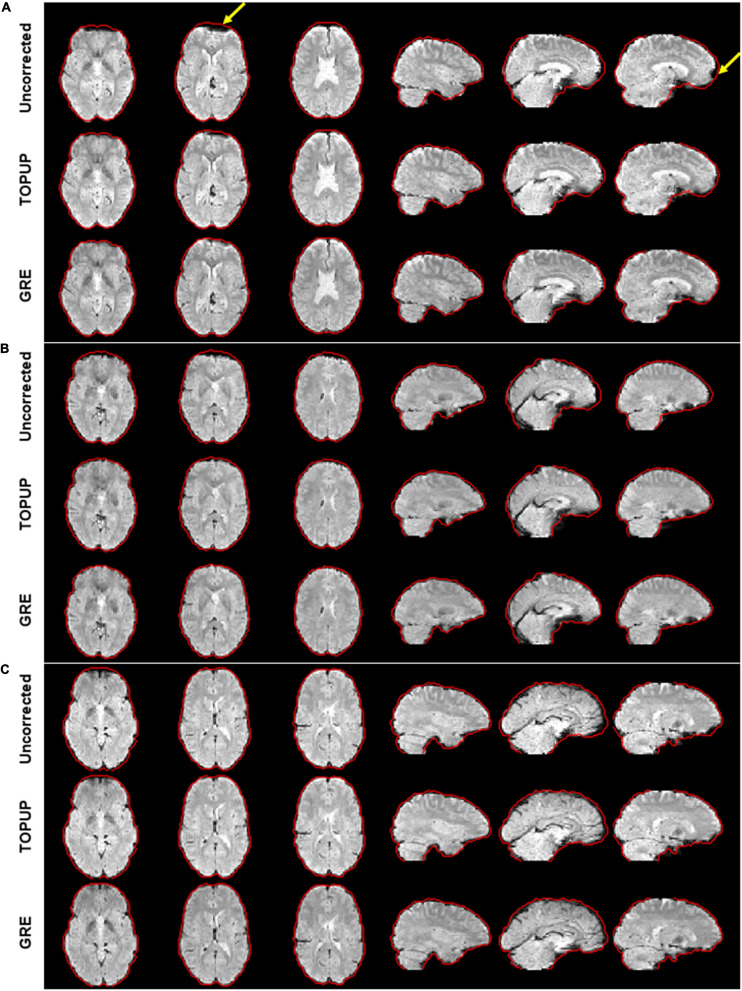
Illustration of the geometric distortions associated with the susceptibility artifact in the fMRI data from three representative participants during the first run of the biological motion perception task. For the first two participants **(A,B)**, distortions highlighted by the yellow arrows can be observed, mostly representing the compression of voxels in areas of tissue/air boundaries. These were highly attenuated after correction with either TOPUP or GRE correction approaches. Participant **(C)** does not present clear distortions. The red traces define the contour of the GRE-corrected images, as this approach yielded the best results, and was then considered here as reference for visualization purposes.

For illustrating the impact of geometric distortions on the fMRI data, examples of the middle volume of the first BM run from three participants are illustrated in Figure 2. Evident compression of voxels at the temporal and frontal lobes, particularly near the air-filled sinuses, can be observed for the first two subjects; the third participant has no apparent geometric distortions, which was uncommonly observed in this study.

The range of ΔB_0_ and voxel shifts in the PE direction, averaged across voxels and participants, are shown in Table 1. Negative voxel shift values correspond to shifts in the anterior-to-posterior direction, whereas positive values indicate shifts in the posterior-to-anterior direction. The GRE approach yields a substantially wider range of voxel shifts (and thus, of ΔB_0_ values) relatively to the TOPUP approach.

**TABLE 1:** Range (minimum and maximum) of ΔB_0_ (in Hz) and voxel shift (in mm) in the phase-encoding direction, averaged across voxels and participants, and across runs.

	ΔB_0_ [Hz] ± std				Voxel shift [mm] ± std			
	
	Min		Max		Min		Max	
	
	TOPUP	GRE	TOPUP	GRE	TOPUP	GRE	TOPUP	GRE
Localizer	−77 ± 13	−163 ± 57	63 ± 17	280 ± 103	−4.3 ± 0.7	−9.1 ± 3.2	3.5 ± 1.0	15.6 ± 5.7
BM 1	−77 ± 12	−168 ± 55	61 ± 17	280 ± 111	−4.3 ± 0.7	−9.3 ± 3.0	3.4 ± 0.9	15.5 ± 6.2
BM 2	−76 ± 12	−174 ± 59	61 ± 17	283 ± 100	−4.3 ± 0.7	−9.7 ± 3.3	3.4 ± 1.0	15.7 ± 5.5
BM 3	−76 ± 13	−169 ± 61	63 ± 18	284 ± 114	−4.2 ± 0.7	−9.4 ± 3.4	3.5 ± 1.0	15.8 ± 6.3
BM4	−74 ± 12	−167 ± 60	58 ± 16	283 ± 120	−4.1 ± 0.7	−9.3 ± 3.4	3.2 ± 0.9	15.7 ± 6.7

**Average**	**−76 ± 12**	**−168 ± 57**	**61 ± 17**	**282 ± 107**	**−4.2 ± 0.7**	**−9.3 ± 3.2**	**3.4 ± 0.9**	**15.7 ± 5.9**

**FIGURE 3:**
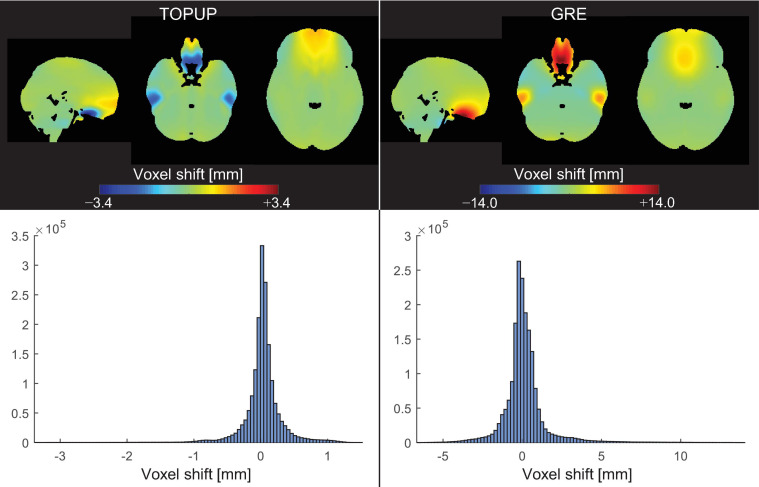
(Top) Voxel shift maps (VSMs), in the MNI space, averaged across participants and runs, for the (left) TOPUP and (right) GRE correction approaches. The scale was set to be symmetric for easing the interpretation of the maps (where green colors always represent no shifts). (Bottom) Histograms of the associated VSMs.

The average VSMs across subjects and runs, in the MNI space, estimated with TOPUP and GRE can be found in Figure 3, together with the respective histograms. Besides the already mentioned difference between the ranges of voxel shifts for the two approaches, it can also be observed that, in the orbitofrontal and temporal regions, TOPUP and GRE diverge regarding the direction of the shifts to be applied. However, the histograms of voxel shifts are quite similar between approaches, despite the rather striking difference in those regions.

**FIGURE 4:**
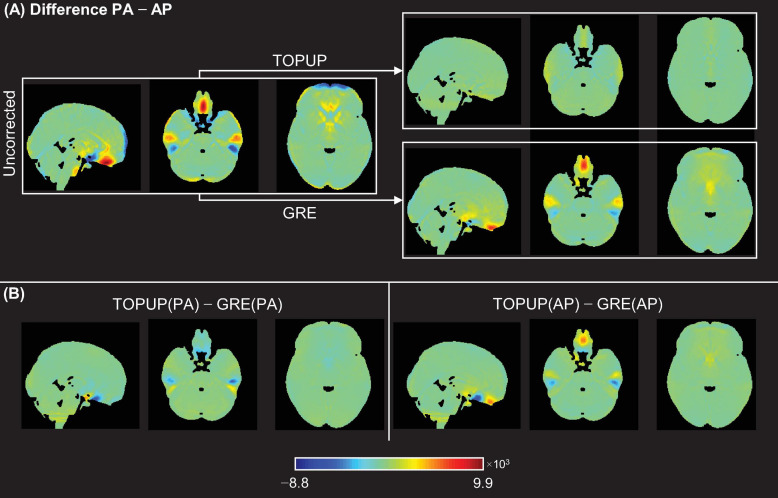
**(A)** Voxel-wise difference maps between PA and AP images for the uncorrected case (left), and after correcting the data for the distortions using TOPUP (top-right) and GRE (bottom-right). **(B)** Voxel-wise difference maps of the corrected PA images (left) and corrected AP images (right), between correction methods.

Next, the difference between the PA and AP images without and with correction using TOPUP and GRE was assessed. The voxel-wise difference maps in the MNI space, averaged across participants and runs, are illustrated in Figure 4A. As expected, TOPUP yields the lowest difference between PA and AP images, as its formulation explicitly determines the displacement field by minimizing it (the map is overall green, corresponding to a difference close to zero). Nonetheless, GRE also approximates the two images, although exhibiting visible differences in the orbitofrontal and temporal regions, but still substantially smaller than the uncorrected case. For investigating the consistency between TOPUP and GRE given their different formulations, the voxel-wise difference maps in the MNI space, averaged across subjects and runs, of the corrected PA and AP images between methods, are shown in Figure 4B. Besides the small differences within the orbitofrontal and temporal regions, the two approaches yield mostly similar PA and AP images (difference maps are overall green).

The nMSE and cross-correlation values from the comparison between PA and AP images (with respect to Figure 4A), averaged across participants (and voxels, in the case of the nMSE), and across runs, are depicted in Table 2. Consistently with what observed in Figure 4A, the *post hoc* analysis revealed that nMSE and cross-correlation statistically significantly decreased and increased, respectively, with the correction for the geometric distortions using both the TOPUP and GRE approaches. As expected, a larger decrease/increase of nMSE/cross-correlation was obtained with TOPUP.

**TABLE 2:** Differences between the PA and AP images without and with distortion correction using TOPUP and GRE, quantified in terms of nMSE and cross-correlation, averaged across participants (and voxels, in the case of the nMSE), and across runs.

	nMSE ± std			Cross-correlation ± std		
	
	Uncorrected	TOPUP	GRE	Uncorrected	TOPUP	GRE
Localizer	0.185 ± 0.019	0.073 ± 0.005	0.126 ± 0.022	0.851 ± 0.026	0.978 ± 0.004	0.925 ± 0.026
BM1	0.187 ± 0.016	0.073 ± 0.004	0.127 ± 0.021	0.847 ± 0.022	0.978 ± 0.004	0.924 ± 0.024
BM2	0.194 ± 0.020	0.074 ± 0.005	0.136 ± 0.027	0.836 ± 0.029	0.977 ± 0.004	0.914 ± 0.033
BM3	0.192 ± 0.018	0.074 ± 0.004	0.135 ± 0.022	0.839 ± 0.026	0.978 ± 0.004	0.915 ± 0.027
BM4	0.191 ± 0.018	0.074 ± 0.005	0.131 ± 0.023	0.842 ± 0.027	0.977 ± 0.005	0.917 ± 0.028

**Average**	**0.190 ± 0.018**	**0.074 ± 0.005**	**0.131 ± 0.023**	**0.843 ± 0.026**	**0.978 ± 0.004**	**0.919 ± 0.028**

The average nMSE and cross-correlation values, across participants (and voxels, in the case of the nMSE), and across runs, of the corrected PA and AP images between correction approaches (with respect to Figure 4B), are presented in Table 3. Low/high values of nMSE/cross-correlation were obtained (<0.13 and >0.92, respectively), evidencing the consistency between TOPUP and GRE at matching the PA and AP images upon correction.

**TABLE 3:** Differences of the corrected PA and AP images between distortion correction approaches quantified in terms of the nMSE and cross-correlation, averaged across participants (and voxels, in the case of the nMSE), and across runs.

	nMSE ± std		Cross-correlation ± std	
	
	PA	AP	PA	AP
Localizer	0.117 ± 0.009	0.119 ± 0.014	0.937 ± 0.009	0.935 ± 0.016
BM1	0.117 ± 0.009	0.120 ± 0.012	0.938 ± 0.009	0.934 ± 0.012
BM2	0.119 ± 0.011	0.130 ± 0.022	0.936 ± 0.012	0.922 ± 0.029
BM3	0.121 ± 0.015	0.129 ± 0.025	0.932 ± 0.018	0.919 ± 0.038
BM4	0.116 ± 0.012	0.124 ± 0.015	0.939 ± 0.012	0.928 ± 0.019

**Average**	**0.118 ± 0.011**	**0.125 ± 0.019**	**0.937 ± 0.013**	**0.927 ± 0.025**

### fMRI Data Analyses

**FIGURE 5:**
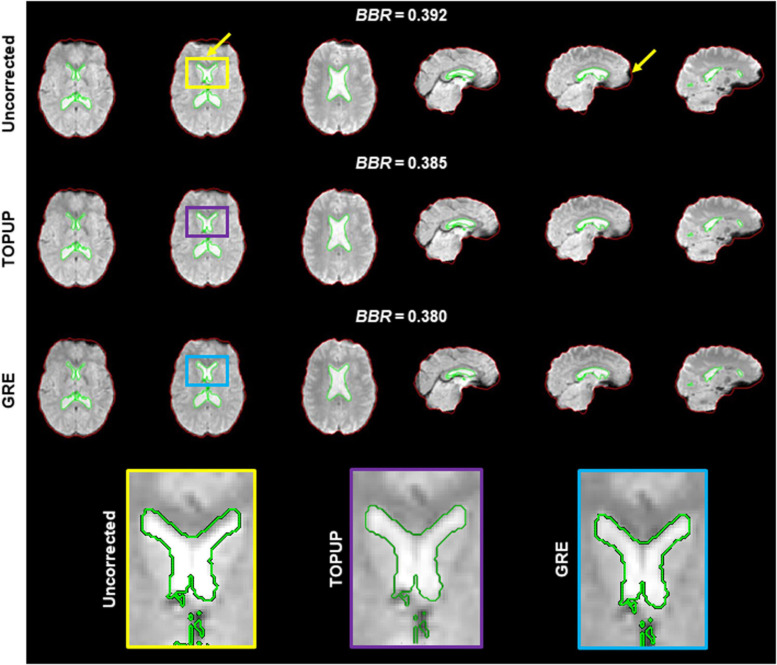
Registration into the structural image of (top) uncorrected, (middle) TOPUP corrected, and (bottom) GRE corrected fMRI data of the first participant illustrated in Figure 2. The red and green traces define the contours of the brain-extracted structural image and its segmented ventricles (I and II), respectively; these can be considered as the ground truth. Besides the squashed voxels in areas of tissue/air boundaries (right yellow arrow), it is clear that the registration of uncorrected data fails to capture the spatial distribution of the ventricles I and II (left yellow arrow); a critical region in ventricle I is zoomed in the yellow, purple and blue boxes. The BBR cost function values are also shown.

Because geometric distortions hinder the accurate registration of functional images into structural images, the BBR cost function values were calculated. Examples of the middle volumes extracted from uncorrected and distortion-corrected fMRI data of the first BM run for the first participant in Figure 2, registered into its structural image, are shown in Figure 5, together with the BBR cost function values. The average across participants for each run (localizer and four BM runs) is shown in Table 4, together with the overall average across runs. For all runs, it is observed a consistent decrease (more accurate registration) in the cost function values when performing geometric distortion correction with TOPUP relatively to no correction, and a further decrease when correcting the distortions with GRE. A significant main effect was observed (*p* = 0.0004), and the cost function values with both TOPUP and GRE corrections were found to be statistically significantly lower than those without correction according to the *post hoc* test.

**TABLE 4:** Average cost function values (across subjects) of the boundary-based registration of the middle volume of each run (localizer and four biological motion runs) into the structural image, for the uncorrected, TOPUP corrected and GRE-corrected fMRI data.

	Correction method		
	
Run	Uncorrected	TOPUP	GRE
Localizer	0.457 ± 0.071	0.447 ± 0.069	0.444 ± 0.071
BM 1	0.460 ± 0.069	0.449 ± 0.068	0.445 ± 0.068
BM 2	0.465 ± 0.071	0.454 ± 0.071	0.451 ± 0.071
BM 3	0.463 ± 0.073	0.451 ± 0.070	0.448 ± 0.071
BM 4	0.467 ± 0.078	0.455 ± 0.077	0.452 ± 0.078

**Average**	**0.462 ± 0.071**	0.452 ± 0.069*	0.448 ± 0.070*


The overall average across runs is also shown. *Indicates statistically significantly (*p* < 0.05) differences between the correction methods and the uncorrected case.

**FIGURE 6:**
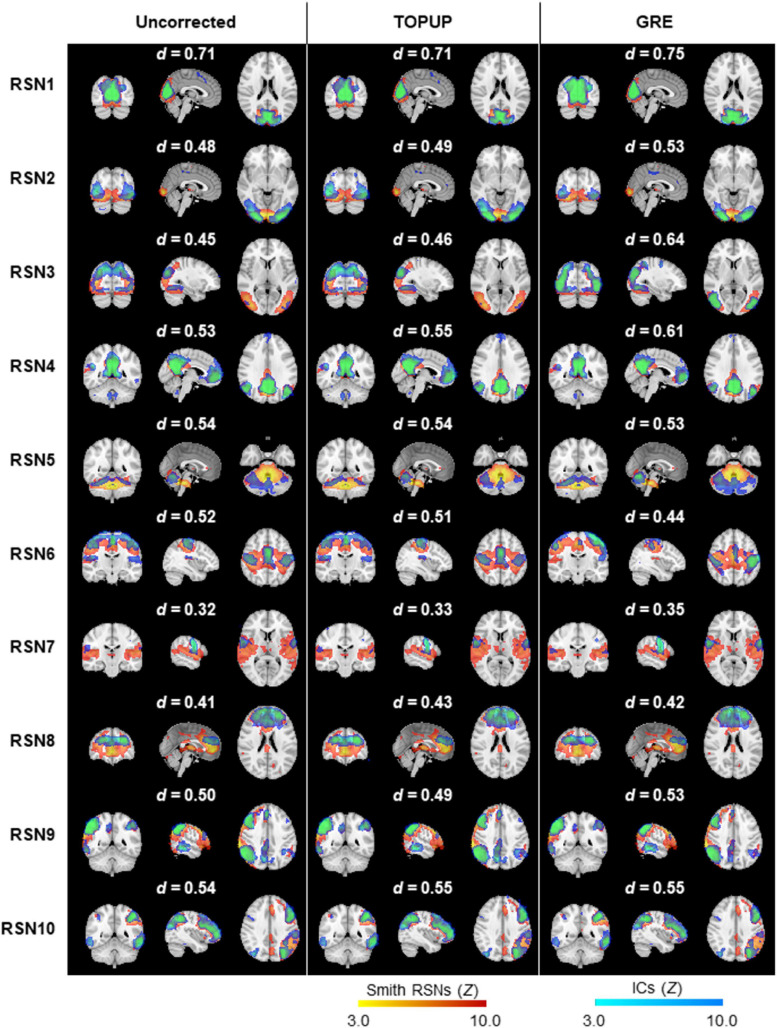
Ten group RSNs identified on (left) uncorrected, (middle) TOPUP corrected, and (right) GRE-corrected fMRI data for the first run of the biological motion perception task. The RSN templates (in red–yellow) from Smith et al. (2009) are superimposed with the spatial independent components (in blue–light blue) selected for each RSN template, according to their Dice coefficient (also shown above each RSN).

Next, we decomposed the fMRI data into ICs using spatial ICA, and identified those associated with RSNs. The group RSNs identified for the first BM run are illustrated in Figure 6, together with the Dice coefficient between them and the respective templates from Smith et al. (2009). It can be observed that the Dice coefficient marginally increased for only six RSNs when comparing the uncorrected with the TOPUP-corrected fMRI data, and maintained or decreased for the remaining four RSNs. This contrasts with the GRE approach, with which a substantial increase in the Dice coefficient values for all RSNs was obtained. The average across RSNs and subjects are depicted in Table 5 for each functional run, as well as the overall average across runs. For the other BM runs, the previous pattern in Figure 6 is even more evident, with TOPUP outperforming the uncorrected case in only one functional run, whereas on average the Dice coefficient values slightly decreased when comparing the uncorrected with the TOPUP-corrected fMRI data. In contrast, correcting the distortions with GRE yielded the highest Dice coefficient values. The ANOVA revealed a notable trend on the main effect of the correction approach, although not reaching statistical significance (*p* = 0.1); nonetheless, the *post hoc* test showed that GRE yielded statistically significantly higher Dice coefficient values than TOPUP.

**TABLE 5:** Average Dice coefficient values (across subjects) quantifying the spatial overlap between the RSN templates from Smith et al. (2009) and the selected group independent components, for each run (localizer and four biological motion runs), and also averaged across runs.

	Correction method		
	
Run	Uncorrected	TOPUP	GRE
Localizer	0.520 ± 0.122	0.516 ± 0.134	0.522 ± 0.129
BM 1	0.509 ± 0.140	0.495 ± 0.145	0.510 ± 0.133
BM 2	0.523 ± 0.121	0.522 ± 0.107	0.524 ± 0.115
BM 3	0.499 ± 0.102	0.504 ± 0.097	0.534 ± 0.117
BM 4	0.501 ± 0.124	0.494 ± 0.124	0.505 ± 0.117

**Average**	**0.510 ± 0.118**	**0.506 ± 0.118**	**0.519 ± 0.118^†^**

*^†^Indicates statistically significant differences (*p* < 0.05) between GRE and TOPUP correction methods.*

The range of voxel shifts within each RSN, from the participant-averaged VSMs in the MNI space, averaged across runs, is shown in Table 6. Consistently with the voxel shift values shown in Table 1, GRE estimated larger shifts overall, which however exhibit a substantial variability across RSNs (also present in the shifts estimated by TOPUP, but to a lesser degree).

**TABLE 6:** Range of voxel shifts, averaged across runs, within each RSN, from the participant-averaged VSMs in the MNI space estimated by TOPUP and GRE.

	Min [mm] ± std		Max [mm] ± std	
	TOPUP	GRE	TOPUP	GRE
RSN1 (Visual)	−1.43 ± 1.18	−2.82 ± 1.36	1.06 ± 0.27	5.28 ± 4.74
RSN2 (Visual)	−1.53 ± 0.71	−3.31 ± 1.57	1.14 ± 0.19	7.41 ± 2.89
RSN3 (Visual)	−1.67 ± 1.13	−4.58 ± 0.98	1.08 ± 0.21	8.95 ± 2.68
RSN4 (DMN)	−1.92 ± 0.73	−3.17 ± 0.58	1.30 ± 0.03	9.03 ± 2.79
RSN5 (Cerebellum)	−1.11 ± 0.78	−4.19 ± 0.25	0.76 ± 0.31	8.49 ± 3.10
RSN6 (Motor)	−1.50 ± 0.57	−3.78 ± 1.47	0.96 ± 0.22	5.71 ± 4.32
RSN7 (Auditory)	−1.29 ± 0.72	−3.61 ± 1.42	0.91 ± 0.31	4.63 ± 1.21
RSN8 (Salience)	−2.18 ± 0.72	−2.94 ± 1.02	1.06 ± 0.18	6.59 ± 1.37
RSN9 (Right Language)	−1.66 ± 0.94	−3.45 ± 1.40	1.03 ± 0.29	5.96 ± 2.23
RSN10 (Left Language)	−2.13 ± 1.02	−2.50 ± 1.25	1.04 ± 0.25	7.61 ± 2.86

**FIGURE 7:**
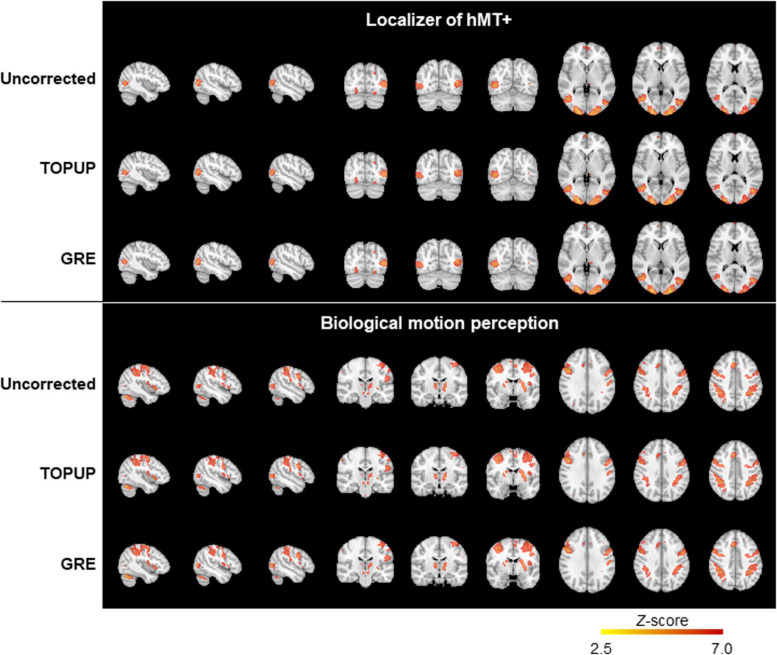
Group activation maps for the localizer (top) and the biological motion (bottom) runs, from uncorrected, TOPUP corrected and GRE-corrected fMRI data.

Finally, we mapped hMT+/V5 from the localizer run, and the brain regions involved in BM perception from the BM runs. The group activation maps (across participants for the localizer run, and participants and runs for the BM runs) are shown in Figure 7. In both cases, the same active regions are highlighted regardless of whether geometric distortions are corrected, or the approach used for their correction. As expected, primary and extrastriate visual areas were activated during the localizer run, including the hMT/V5 area. Regions involved in BM perception included: the posterior part of the superior temporal sulcus, the inferior frontal gyrus, the superior parietal lobe, and sub-cortically the insula and the thalamus; the cerebellum was also activated. The average and maximum *Z*-score values of each group activation map are depicted in Table 7. Consistent with the previous visual inspection, marginal differences can be observed regarding the average *Z*-score across the distortion correction approaches, and the uncorrected case (*p* > 0.05). In contrast, a clear trend was found (*p* = 0.06) regarding the differences in the maximum *Z*-score values, that followed the same pattern of the BBR cost function values: a consistent increase from the uncorrected to the TOPUP-corrected fMRI data, and a further increase when performing the GRE correction. This trend was confirmed by the *post hoc* test, with the GRE approach yielding statistically significantly higher maximum *Z*-score values than the uncorrected case. For both the localizer and BM runs, and for the uncorrected and TOPUP- and GRE-corrected cases, the voxel of maximum *Z*-score was located at V1.

**TABLE 7:** Average $\left(\bar{\text{z}}\right)$ and maximum (z_max_) Z-score values of the group activation maps for the localizer and biological motion runs. The overall average across runs is also shown.

	Correction method $\left(\bar{\text{z}}|{\text{z}}_{\max}\right)$		
	
Run	Uncorrected	TOPUP	GRE
Localizer	3.99 ± 0.74 | 6.85	4.01 ± 0.75 | 7.11	4.02 ± 0.76 | 7.23
BM	4.19 ± 0.53 | 6.85	4.19 ± 0.54 | 6.94	4.20 ± 0.54 | 7.10

**Average**	**4.09 ± 0.64 | 6.85**	**4.10 ± 0.65 | 7.03**	4.11 ± 0.65 | 7.17*

*Indicates statistically significant (*p* < 0.05) differences between the maximum *Z*-score values obtained with GRE and those from the uncorrected case.

Similarly to the RSN analysis, the minimum and maximum voxel shifts within each activation map (for the localizer and BM tasks), were extracted from the participant-averaged VSMs in the MNI space. For the localizer run, the minimum/maximum voxel shifts were −0.41/0.51 and −0.38/2.77 mm for the TOPUP and GRE, respectively; for the BM run, the minimum/maximum voxel shifts were −0.63/0.95 and −2.31/5.01 mm for the TOPUP and GRE, respectively. Consistently, GRE estimated the largest shifts, albeit substantially smaller than those obtained within some of the RSNs.

## Discussion

In this study, we have characterized the geometric distortions and the correction approaches based on the estimated ΔB_0_ field offset and voxel shift maps, and quantitatively assessed the impact of geometric distortions on several fMRI data analyses. We directly compared, in the same dataset, two different approaches of distortion correction and their impact on data analyses, including the registration of functional images into structural images, the identification and characterization of RSNs, and the mapping of regions of interest during tasks involving the simple perception of motion, and the more complex visual perception of biological motion.

### Impact of Geometric Distortions on fMRI Data Analyses

We started by quantifying the registration quality between the functional and the structural images with the BBR cost function values, and found that correcting geometric distortions with both TOPUP and GRE approaches improved the registration, both yielding significantly lower cost function values relatively to registering uncorrected functional images, and with GRE exhibiting the best performance. This observation was expected because the registration quality has been the most explored metric in the literature to assess the impact of geometric distortions on both fMRI and diffusion tensor imaging (DTI) data, and its correction approaches, with most studies pointing toward more accurate registrations upon distortion correction (Jezzard and Balaban, 1995; Hutton et al., 2002; Andersson et al., 2003; Ardekani and Sinha, 2005; Holland et al., 2010; Bhushan et al., 2015; Glodeck et al., 2016; Graham et al., 2017).

Interestingly, very few studies went beyond measuring the registration quality and investigated the extent at which geometric distortions impact fMRI data analyses, without however comparing distortion correction approaches. Besides the registration quality and the amount of voxel shifts across the brain, the first study also focused on the statistical power of conventional group-level analyses of fMRI data collected during auditory and motor tasks (Cusack et al., 2003). While for the auditory task the correction of geometric distortions with a GRE-based approach increased the extent and the overall *Z*-scores of the group activation map relatively to that obtained without correction, voxels in the primary motor areas were only active during the motor task upon correcting the fMRI data for the distortions. For our two tasks (moving dots for functionally localizing hMT+/V5 and visual BM perception), the group activation maps obtained with and without distortion correction, and across correction approaches, were indistinguishable; nonetheless, the statistical power (based on the maximum *Z*-score values) of the group-level analyses increased with distortion correction, particularly using the GRE approach with which a statistically significant increase was obtained; no differences were found for the average or maximum *Z*-score values when considering the single subject activation maps. These changes in the *Z*-score values may be associated with the more accurate registrations (i.e., lower BBR cost function values) obtained with TOPUP, and especially GRE, relatively to the uncorrected case, decreasing the presence of unrealistic additional variability in the group-level analyses, which in turn increases their statistical power. In contrast with TOPUP, GRE estimated modest, but non-zero, voxel shifts within the regions involved in these tasks, which may additionally explain the small increase, albeit significant, in the maximum *Z*-score values, which was not found when considering TOPUP. The small voxel shifts estimated by both approaches, especially TOPUP, suggest that these task-related regions are not particularly prone to geometric distortions, representing a limitation of this fMRI analysis. Importantly, the voxels with maximum *Z*-score were located at V1 in all analyses, suggesting that this measure is robust and physiologically meaningful. The fact that only changes in the *Z*-score values, rather than the extent of the activation, were observed suggests that by correcting the geometric distortions, only the sensitivity, rather than the specificity, was impacted. In our case, this is an important result since the regions of interest during both tasks were already highlighted when analyzing the uncorrected fMRI data, and potentially extending them with the correction of the distortions would result in an undesired loss in specificity.

How RSNs are affected by geometric distortions has been more recently investigated (Togo et al., 2017). Similarly to our study, a sub-set of four RSNs comprising brain regions more susceptible to distortions were identified with spatial ICA, and their intra- and inter-network functional connectivity were estimated. Specifically, the most substantial increase in intra-network connectivity was observed for the DMN when correcting the fMRI data for the distortions with a GRE-based approach. Here, we broaden this analysis to the ten most commonly studied RSNs in the literature, which included the DMN and other RSNs that in principle would not be substantially impacted by the distortions. We independently validated, and assessed the quality of, their identification based on the overlap (quantified by the Dice coefficient) with well-recognized RSN templates (Smith et al., 2009). An overall increase in the overlap was observed when correcting the distortions with the GRE approach, irrespective of the RSN, which yielded significantly higher Dice coefficient values than those obtained with the TOPUP approach. The largest increase in overlap (difference higher than 0.05) with distortion correction was observed for the RSNs 3 (visual), 4 (DMN), and 10 (left language). Consistently, the largest absolute voxel shifts (>7.50 mm) estimated with GRE were also found within these three networks, and additionally the RSN 5 (cerebellum), suggesting that the identification of the most distorted networks, upon correction with larger voxel shifts, improved more visibly, which thus supports the validity of this metric for quantifying the impact of geometric distortions. Importantly, besides the DMN, which is consistent with the results from Togo et al. (2017), the left language network also comprises regions typically affected by distortions, namely the frontal and temporal lobes (Hutton et al., 2002), thus supporting the relevance of correcting geometric distortions particularly when focusing the analyses on such sensitive brain regions. Although RSN templates cannot be regarded as ground truth for RSNs, they have been extremely useful for identifying RSNs (here and in several previous studies), and assessing changes in their spatial maps with distortion correction.

### Comparison of Different Geometric Distortion Correction Approaches

From the comparison of the averaged VSMs estimated by each correction approach, we found that they were distinct, mainly in the temporal and orbitofrontal regions, where TOPUP and GRE diverge regarding the direction of the shifts to be applied (anterior-to-posterior and posterior-to-anterior, respectively), and with GRE estimating larger shifts. Such smaller shifts estimated by TOPUP may partially be explained by its formulation that uses the actual functional PA and AP images (rather than undistorted GE images as in GRE) which, in this case, were acquired using 2× GRAPPA in-plane acceleration, thus reducing the amount of distortions by shortening the time between phase-encoding steps. Despite this rather striking difference of the VSMs at these specific brain regions, the associated histograms, however, were quite similar between approaches. This is in line with the similarity of the corrected PA and AP images, between correction approaches. This was quantified in terms of the nMSE and cross-correlation, and we found that, for all runs (and on average across runs), low/high values of nMSE/cross-correlation were obtained (<0.13 and >0.92, respectively), evidencing the consistency between the two approaches, and thus partially justifying the rather small differences, albeit statistically significant in some cases, observed in the fMRI data analyses. Importantly, we also assessed the difference between PA and AP images after correcting them with TOPUP and GRE, and without correction, and found that the nMSE and cross-correlation values significantly decreased and increased, respectively, with distortion correction regardless of the approach, although more evident with TOPUP. Such result supports the significant impact of distortion correction in some of the fMRI data analyses, while showcasing the ability of GRE to appropriately approximate the PA and AP images even if its formulation is not explicitly designed at such as that of TOPUP is.

Next, we focused on quantifying the impact of geometric distortions and their correction with TOPUP and GRE on several fMRI data analyses. Only a number of studies have also conducted such systematic comparison, but none focused on fMRI data. From data collected at 7T, which is more sensitive to B_0_ field inhomogeneity, measurements of the T_1_ constant and brain perfusion were performed and their quality investigated as a function of several distortion correction approaches (Hong et al., 2015). Non-linear registration, field mapping (GRE) and reversed phase-encoding (TOPUP) were tested; GRE and TOPUP significantly improved the quantification of the measures of interest, with TOPUP outperforming GRE. These results are in agreement with a recent DTI simulation study (Graham et al., 2017), whereby diffusion data were simulated with and without geometric distortions, the latter defining the ground truth to be recovered from the distorted data. Distortions were corrected using the abovementioned approaches, and multiple metrics extracted to quantify its impact, namely the voxel displacement and intensity maps, and several diffusion measures. Consistently with (Hong et al., 2015), non-linear registration yielded the poorest results, and the TOPUP approach outperformed the GRE approach. In agreement with these previous studies, our results show that correcting the fMRI data for distortions positively impacted its subsequent analyses. Despite the GRE approach, rather than the TOPUP approach, yielded the best results in our case, the latter was also found to improve the quality of most analyses performed. This may also be associated with the range of voxel shifts estimated from each approach, and the amount of spatial smoothing applied. In fact, while the range of voxel shifts estimated from TOPUP (−5 to 4 mm, approximately) closely matched the spatial smoothing kernel of 4 mm used here, those from GRE (−10 to 16 mm, approximately) clearly surpassed it. Nonetheless, correcting distortions on regions where larger voxel shifts need to be applied (and thus, where geometric distortions are more severe) will be critical regardless of the spatial smoothing step, especially when considering GRE which estimated the largest voxel shifts.

Importantly, here TOPUP was applied to GE-EPI images with reversed phase-encoding directions, rather than spin-echo (SE)-EPI images, for which TOPUP was initially designed for. The core difference between the two imaging sequences lies in the application of an additional 180° refocusing pulse at TE/2 in SE-EPI, preventing through-plane dephasing (i.e., dephasing orthogonal to the imaging plane) which would result in the signal dropouts that are present in GE-EPI images (Embleton et al., 2010; In et al., 2015). Therefore, these dropouts are independent from the acquisition scheme of the *k*-space (and hence, the imaging plane), rendering the formulation of TOPUP still valid for estimating the displacement fields on GE-EPI images even in the presence of dropouts. This has been hypothesized by the original authors of TOPUP in Andersson et al. (2003), and confirmed in multiple, and recent fMRI studies at 3T using data collected during task performance (Shan et al., 2018) and rest (Disner et al., 2018), and especially in studies at 7T (Emmerling et al., 2016; Kashyap et al., 2018; Bréchet et al., 2019) where distortions (and signal dropouts) are more severe. Nonetheless, it has been suggested that SE-EPI-derived displacement field maps applied to GE-EPI images render the most accurate distortion correction, because the estimated fields would not be affected by signal dropouts (Holland et al., 2010; Glasser et al., 2016). However, this has never been explicitly tested, and other factors should be considered, particularly the differences in SE-EPI and GE-EPI image intensities and the less critical longer scanning times for SE-EPI. In fact, acquiring GE-EPI images with a reversed phase-encoding direction is extremely fast (one TR per image), and because TOPUP approaches have the ability to account for (less critical) pixel intensity variations (which lacks in GRE-based approaches), their use on GE-EPI images may be a potentially powerful correction approach. Nonetheless, more complex approaches able to capture the dynamic aspects of distortions can be considered. In fact, conventional correction approaches typically assume that a single estimated displacement field is valid for the whole scanning session, or may estimate a displacement field per fMRI run, as it was done here. Even in the latter case, considering that one displacement field is representative of the distortions at all brain volumes acquired in an fMRI run is intrinsically assuming that no head motion has occurred between the acquisition of the field map and the fMRI data, and neither during the fMRI run, since head motion (specifically out-of-plane rotations) is known to non-linearly interact with geometric distortions (Hutton et al., 2002; Jezzard, 2012). This represents a limitation of most distortion correction approaches, including the ones used here, which has been circumvented by embedding the acquisition of continuous field map data into the fMRI acquisition (Roopchansingh et al., 2003; Weiskopf et al., 2005), or by retrospectively computing volume-specific field maps from a single field map and the head motion parameters of the associated fMRI data (Yeo et al., 2008; Takeda and Kim, 2013). Because of the increased scanning time of the former, and the loss in computational efficiency and simplicity of the latter, approaches explicitly tackling the interaction of head motion with geometric distortions are still seldom used. Coupling these considerations with the low head motion observed here, we believe that such complex approaches may not change the results and respective conclusions.

### Minimizing a Priori Geometric Distortions

Besides avoiding head motion through appropriate head fixation systems, the susceptibility artifact can be minimized using PI, which reduces the number of phase-encoding steps. PI can be applied in the image space or in *k*-space, the latter being the one used in this study (specifically GRAPPA). By using GRAPPA (although with a modest acceleration factor of 2), the susceptibility artifact in our data was expected to be less prominent, and thus may partially justify the notable, but not statistically significant, differences in some fMRI analyses. A recent debate on whether in-plane and SMS accelerations should be employed together has emerged. A first study systematically investigated the sensitivity and false-positive activation of analyses of fMRI data collected at 3T using different SMS factors, and suggested that a conservative combination of 2× GRAPPA with 2× SMS accelerations yields fMRI data with modest geometric distortions and without apparent slice leakage (Todd et al., 2016); this is in line with the acquisition parameters used in this study.

## Conclusion

In this study, we have characterized the geometric distortions and the correction approaches based on the estimated ΔB_0_ field offset and voxel shift maps, and quantified the impact of geometric distortions and their correction by two approaches (TOPUP and GRE) for the estimation of the underlying displacement field, on the quality of conventional fMRI data analyses. We showed that accounting for geometric distortions in fMRI data is recommended for this specific application, with TOPUP and GRE estimating distinct VSMs (mainly locally), and that the choice of the approach had a modest, albeit positive, impact on all fMRI analyses. In particular, GRE achieved statistically significant improvement for the registration between the functional and structural data, and the sensitivity of the mapping of task-related regions of interest, while TOPUP only yielded significant improvements for the registration analysis. Importantly, the additional data necessary for GRE required a substantially longer scanning time (∼3.5 min) than that for TOPUP (10 s, 1 volume per TR), which may present a limitation. Future studies, with larger datasets collected using different experimental protocols and setups will be needed to reproduce the conclusions claimed here, which were drawn from this first study directly comparing, in the same dataset, two different geometric distortion correction approaches on fMRI data analyses.

## Data Availability Statement

The raw data supporting the conclusions of this article will be made available by the authors, without undue reservation.

## Ethics Statement

The studies involving human participants were reviewed and approved by Comissão de Ética da Faculdade de Medicina da Universidade de Coimbra. The patients/participants provided their written informed consent to participate in this study.

## Author Contributions

Both authors coded the stimulus presentation and acquired the data. RA performed the data analyses and wrote the manuscript. JD reviewed the manuscript.

## Conflict of Interest

The authors declare that the research was conducted in the absence of any commercial or financial relationships that could be construed as a potential conflict of interest.

## Footnotes

1 https://fsl.fmrib.ox.ac.uk/fsl/fslwiki/MELODIC
